# Enhancement of Phenolic Content and Antioxidant Capacity in Chiffon Cake Through Partial Replacement of Low-Gluten Flour with Mycoprotein Powder

**DOI:** 10.3390/foods15010116

**Published:** 2025-12-31

**Authors:** Kuo-En Cheng, Li-Yun Lin, Chin-Chu Chen, Jia-Hsin Guo

**Affiliations:** 1 Department of Food Science, College of Agriculture, National Pingtung University of Science and Technology, Neipu, Pingtung 912301, Taiwan; cheng9104@hk.edu.tw; 2 Department of Food Science and Technology, College of International Hospitality and Tourism, Hungkuang University, Shalu District, Taichung 433304, Taiwan; 3 Biotech Research Institute, Grape King Bio Ltd., Long Tan District, Taoyuan 325002, Taiwan; gkbioeng@grapeking.com.tw; 4 Institute of BioPharmaceutical Science, National Sun Yat-Sen University, Kaohsiung 804201, Taiwan; 5 Institute of Food Science and Technology, National Taiwan University, Taipei 106319, Taiwan

**Keywords:** mycoprotein, *Fusarium venenatum*, chiffon cake, free and bound phenolics, flavonoid content, antioxidant capacity, fungal protein, functional bakery product

## Abstract

The chiffon cake is widely consumed and is especially age-friendly due to its soft texture and ease of swallowing. However, conventional formulations based on low-gluten flour (LGF) are low in bioactive compounds and offer limited nutritional value. This study examined whether partial replacement of LGF with mycoprotein powder (MPP), a sustainable fungal protein rich in phenolics and flavonoids, could enhance the functional properties of chiffon cakes, with substitution levels up to 80% of LGF. Building on our previous work showing that mycoprotein substitution preserves chiffon cake texture and sensory quality, we focused on phenolic composition and antioxidant capacity in this study. Free and bound phenolics were quantified directly in cake samples, and bioactives were extracted with water, 50% ethanol, and 95% ethanol. Total flavonoid content and antioxidant capacity were assessed using DPPH, ABTS, ferric reducing antioxidant power, ferrous-ion chelating activity, and reducing power assays. Increasing MPP levels significantly elevated extraction yield, total phenolics, and total flavonoids, with 50% ethanol giving the highest recoveries. At 80% MPP substitution, total phenolics nearly doubled, and flavonoids increased more than six-fold, accompanied by marked improvements across all antioxidant assays. These findings support mycoprotein fortification as a viable strategy to enhance the nutritional and functional quality of chiffon cakes.

## 1. Introduction

Global interest in sustainable and nutritionally dense protein sources has intensified in response to population growth, resource limitations, and increasing awareness of environmental impacts. Alongside plant-based dietary trends, microbial proteins have emerged as promising alternatives due to their efficient production cycles, minimal agricultural input, and favorable nutritional profiles. Mycoprotein, a filamentous fungal biomass produced through controlled fermentation, exemplifies this potential. The best-known commercial example is *Fusarium venenatum*-derived Quorn™, which has been marketed since the 1980s in Europe and North America. Mycoprotein is classified as “Generally Recognized as Safe” (GRAS) by the United States Food and Drug Administration and is valued for its high protein content, dietary fiber, and low-fat levels, making it an attractive ingredient for next-generation food formulations [1,2].

Beyond macronutrient advantages, mycoprotein contains diverse secondary metabolites, including phenolic acids, polyphenols, flavonoids, terpenoids, and alkaloids, which collectively contribute antioxidant, antimicrobial, and anti-inflammatory functions [3,4]. Many of these compounds can scavenge reactive oxygen species (ROS), donate electrons, or chelate pro-oxidant metal ions, thereby supporting oxidative stability in foods and potentially benefiting human health [5,6]. Because thermal processing often reduces endogenous antioxidant compounds in bakery systems, ingredients that retain or deliver active phenolics during baking are increasingly sought for functional bakery applications. Studies incorporating mushroom-derived powders into bread matrices have reported significant increases in total phenolic content (TPC) and improved antioxidant indicators such as 2,2-diphenyl-1-picrylhydrazyl (DPPH) scavenging and ferric reducing antioxidant power (FRAP) [7,8]. Mycoprotein shares taxonomic and biochemical characteristics with edible fungi and contains phenolic acids such as gallic acid and *p*-hydroxybenzoic acid, which exhibit strong radical quenching and reducing capacities [9].

Structural characteristics of fungal biomass further enhance its relevance in baked systems. The β-glucan and chitin rich cell wall of *F. venenatum* can entrap phenolic compounds, many of which occur in bound form and are released through alkaline hydrolysis, thermal processing, or mechanical disruption [10]. Such mechanisms are particularly pertinent in chiffon cake, where heating and aeration may liberate cell wall-associated phenolics and increase extractable antioxidant content.

The chiffon cake is widely consumed due to its soft texture and ease of digestion, making it particularly suitable for children and older adults. However, its primary ingredient, low-gluten wheat flour, contributes limited bioactive compounds. Incorporating mycoprotein powder (MPP) into chiffon cakes therefore represents a practical strategy to enrich phenolic and flavonoid content while simultaneously enhancing overall antioxidant potential. Prior studies have demonstrated strong antioxidant behavior in *F. venenatum* extracts, including 50–60% inhibition of DPPH radicals at 0.25 mg/mL in acetonitrile and methanol fractions [11], as well as improvements in reducing power (RP) and radical scavenging capacity in baked goods fortified with fungal or mushroom-derived powders [12,13].

Our previous study systematically characterized the effects of mycoprotein substitution on batter rheology, texture profile, and sensory acceptability of chiffon cakes, showing that moderate substitution levels preserved desirable sensory attributes [14]. Building on that work, we systematically examined how graded substitution of low-gluten flour (LGF) with MPP affects phenolic composition and antioxidant capacity in chiffon cake, with particular emphasis on free and bound fractions and multiple antioxidant mechanisms. Free and bound phenolics were quantified to determine TPC, providing insight into both readily extractable and structurally bound phenolic fractions within the cake matrix. Extraction yield and total flavonoid content (TFC) were assessed using solvents of differing polarity to characterize the solubility and distribution of bioactive constituents. Antioxidant activity was evaluated using multiple complementary assays, including DPPH and 2,2′-azino-bis-(3-ethylbenzothiazoline-6-sulfonic acid) (ABTS) radical-scavenging, FRAP, ferrous ion-chelating activity (FICA), and RP.

We hypothesize that incorporating mycoprotein powder into chiffon cakes increases extractable phenolic content, elevates total flavonoids, and enhances antioxidant performance across radical-scavenging, electron-transfer, and metal-chelating assays. By integrating compositional, extraction based, and functional analyses, this study aims to establish mycoprotein as a dual function ingredient that contributes both high quality protein and natural antioxidants, supporting the development of chiffon cakes with enhanced nutritional and functional value for children, older adults, and health-conscious consumers.

## 2. Materials and Methods

### 2.1. Experimental Materials

Food-grade ingredients for mycoprotein chiffon cakes (MPCs) were obtained from the same suppliers as in our previous study [14]. Briefly, LGF (Top Food Industry Corporation, Taichung, Taiwan) and *F. venenatum*-based MPP (Grape King Bio Ltd., Taoyuan, Taiwan) were the principal dry ingredients; whole eggs (Tainung Egg Products Co., Ltd., Kaohsiung, Taiwan), fine granulated sugar (Taiwan Sugar Corporation, Tainan, Taiwan), baking powder (Wan Chee Trading Co., Ltd., Taipei, Taiwan), refined soybean oil (Taiwan Sugar Corporation, Tainan, Taiwan), food-grade rum (Taiwan Tobacco & Liquor Corporation, Taipei, Taiwan), and isomalto-oligosaccharide syrup (Sweet Town Enterprise Co., Ltd., Hualien, Taiwan) completed the formulation. Rum was included to mitigate mushroom-like notes from MPP and reduce eggy odor, as established in our prior work [14].

All chemicals were of analytical grade and purchased from Sigma-Aldrich (St. Louis, MO, USA). Solvents for extraction and assays, ethanol and methanol, were of HPLC grade (Sigma-Aldrich). Deionized water produced using an Elix^®^ Essential Water Purification System (RiOs Essential, Wild Blue Co., Ltd., Hsinchu, Taiwan) was used for all aqueous preparations.

### 2.2. Preparation of MPC Samples

MPCs were prepared in the laboratory pilot kitchen of the Department of Food Science following the formulation and baking procedures described by [14], with minor modifications. The detailed recipe, including the mixing ratios of LGF and MPP for each formulation (MPC0, MPC20, MPC40, MPC60, and MPC80), as well as the proximate composition of all ingredients, is summarized in Table 1. For each formulation, the required amounts of LGF and MPP were weighed, thoroughly blended, and sifted together with baking powder to obtain a homogeneous flour mixture prior to batter preparation.

All batter preparations were carried out under controlled conditions using a standardized procedure to ensure batch-to-batch consistency. First, the liquid ingredients (soybean oil, water, rum, and sugar (1)) were combined in a stainless-steel mixing bowl and stirred until the sugar was fully dissolved and a homogeneous mixture was obtained. The pre-sifted flour mixture was then gradually added to the liquid in several portions while mixing manually with a wire-whisk mixer. Mixing was continued until a smooth, lump-free base batter was formed, taking care to avoid overmixing that could adversely affect gluten development and batter viscosity.

In parallel, a meringue phase was prepared. Egg whites were transferred to a clean, grease-free bowl and whipped using a Dynasty gear-driven desktop mixer (HL-11007, Hsiao Lin Machine Co., Ltd., Taichung, Taiwan). Isomalto-oligosaccharide and sugar (2) were added gradually during whipping. Whipping continued until the egg whites reached a semi-stiff peak stage, close to a dry foam, characterized by peaks that stood upright with only slight bending at the tips. This endpoint was chosen to obtain a stable meringue capable of entrapping air and supporting the characteristic foam structure of chiffon cakes.

The final batter was obtained by folding the meringue into the flour-based batter in three portions. Approximately one-third of the meringue was first added to the base batter and gently incorporated using a rubber spatula with a circular and “cut-and-fold” motion to lighten the batter. The remaining meringue was then folded in two additional portions, each time rotating the bowl and lifting the batter from the bottom to the top to ensure uniform distribution while minimizing air loss. Folding was continued only until no visible streaks of meringue remained and the batter appeared homogeneous and aerated.

For each formulation, 325 g of the finished batter was weighed into an ungreased 6-inch (approximately 15 cm diameter) chiffon cake pan (tube-type aluminum mold). The surface of the batter was gently leveled with a spatula, and the pan was tapped lightly on the bench to release any large air bubbles. Cakes were baked in a preheated K-COMPACT high-quality mini professional oven (K-35-IAB-S, Chung Pu Baking Machinery Co., Ltd., Taichung, Taiwan) at 190 °C (upper heat) and 130 °C (lower heat) for 45 min on the middle rack to promote uniform heat distribution. At the end of baking, the pans were removed from the oven and immediately inverted on a wire rack to cool at room temperature (25 ± 2 °C), allowing the cakes to set without collapsing.

After complete cooling, the cakes were carefully removed from the pans. Only the inner crumb portion was collected for analysis; the crust and outer browned layer were trimmed away to minimize variability arising from surface browning and moisture gradients. Each formulation was baked in triplicate on separate days, and analytical measurements were performed in triplicate (*n* = 3) using crumb samples taken from the central region of each cake to ensure sample uniformity.

### 2.3. Moisture Content

The moisture content of chiffon cake samples was determined by oven drying. Approximately 1–2 g of sample (*W*_1_) was weighed into a clean, pre-dried weighing bottle of known constant weight (*W*). The bottle containing the sample was dried in a hot-air oven at 105 °C under atmospheric pressure until a constant weight was reached, and the final weight of the bottle plus dried sample was recorded as *W*_2_. Moisture content was calculated from the loss in mass during drying using the following equation:

(1) $$Moisture\;Content\left(\%\right)=\;\left[W_{1}-(W_{2}-W\right)/W_{1}]\times 100,$$

where *W* is the mass of the empty weighing bottle, *W*_1_ is the initial mass of the sample, and (*W*_2_ − *W*) is the mass of the dried sample.

### 2.4. Determination of TPC

#### 2.4.1. Free Phenolic Content

Free phenolic compounds were quantified following the method of [15] with minor modifications. Briefly, 0.5 g of oven-dried and finely crumbled MPC sample was mixed with 8 mL of 80% (*v*/*v*) methanol and subjected to ultrasonic extraction at 35 °C for 1 h. An additional 8 mL of 80% methanol was then added, followed by a second 1 h ultrasonic extraction under the same conditions. The mixture was centrifuged at 5000 rpm for 10 min using a refrigerated centrifuge (Eppendorf Centrifuge5427 R, Eppendorf AG, Hamburg, Germany), and the supernatant was collected. The pH of the collected extract was adjusted to 4.5–5.5 using 6 M HCl to obtain the working solution.

Free phenolics were quantified using the Folin–Ciocalteu colorimetric method. Standard solutions of gallic acid (0–600 μg/mL) were prepared to generate a calibration curve. The calibration curve was linear over this range with R^2^ = 0.9976. For analysis, 100 μL of sample extract or standard solution was combined with 400 μL of deionized water and 100 μL of the Folin–Ciocalteu reagent. After a 6 min reaction, 1 mL of 7% (*w*/*v*) Na_2_CO_3_ and 0.8 mL of deionized water were added. The mixture was incubated at room temperature for 90 min, and absorbance was measured at 760 nm using a UV-Vis spectrophotometer (UV-2600, Shimadzu, Kyoto, Japan). Free phenolic content was calculated from the standard curve and expressed as μg gallic acid equivalent (GAE) per gram of sample (μg GAE/g; dried basis).

This colorimetric protocol and the expression of results as gallic acid equivalents are consistent with recent work on phenolic-rich food systems, including nanophytosomes encapsulating pomegranate fruit extract [16].

#### 2.4.2. Bound Phenolic Content

Bound phenolics were analyzed from the residue remaining after free phenolic extraction, using the method of [15] with modifications. The residue was rinsed with 20 mL of deionized water and centrifuged to remove excess moisture. The pellet was mixed with 20 mL of 4 M NaOH and ultrasonically extracted at 35 °C for 2 h. A second 20 mL aliquot of 4 M NaOH was added and the mixture centrifuged at 5000 rpm for 10 min. The resulting supernatant was collected, and its pH was adjusted to 4.5–5.5 with 6 M HCl.

Bound phenolics were quantified using the same Folin–Ciocalteu procedure described above. Gallic acid standards (0–600 μg/mL) were used to prepare the calibration curve. The calibration curve was linear over this range with R^2^ = 0.9976. For each reaction, 100 μL of sample hydrolysate or standard solution was combined with 400 μL of deionized water and 100 μL of the Folin–Ciocalteu reagent. After 6 min, 1 mL of 7% Na_2_CO_3_ and 0.8 mL of water were added, followed by a 90 min incubation at room temperature. Absorbance was measured at 760 nm, and bound phenolics were calculated from the gallic-acid standard curve and expressed as μg GAE/g (dried basis).

#### 2.4.3. Calculation of TPC

TPC was calculated as the sum of free phenolics and bound phenolics and expressed as μg GAE/g (dried basis):

(2) TPC=Free Phenolics+Bound Phenolics

### 2.5. Extraction of Bioactive Compounds

The extraction procedure for bioactive compounds was adapted from [17] with modifications to suit the chiffon cake matrix. After cooling to room temperature, the inner crumb of each MPC formulation was gently crumbled and oven-dried to obtain a uniform sample. For each solvent extraction, 1.00 g of the crumbled cake was weighed into a 50 mL centrifuge tube and combined with 10.0 mL of one of three solvents representing a polarity gradient: deionized water (high polarity), 50% ethanol (intermediate polarity), or 95% ethanol (low polarity). Samples were subjected to ultrasonic-assisted extraction for 2 h in a temperature-controlled water bath maintained at ≤30 °C to minimize thermal degradation of phenolic and antioxidant compounds. After the initial extraction, an additional 10.0 mL of the same solvent was added, followed by a second sonication step for 1 h to maximize compound recovery.

The extracts were filtered through Whatman No. 4 filter paper (GE Healthcare Life Sciences, Marlborough, MA, USA). Ethanolic filtrates were concentrated to dryness under reduced pressure at 65 °C, whereas aqueous filtrates were concentrated at 85 °C. The dried extract weight (DEW) of each sample was recorded, after which the dried residues were reconstituted to a final volume of 10.0 mL with their corresponding extraction solvent. All reconstituted extracts were passed through a 0.45 µm membrane filter, transferred to clean centrifuge tubes, and stored under refrigeration (≤4 °C) until analysis of TFC and antioxidant activities. Unless otherwise noted, TFC and antioxidant indices are expressed per gram of dry cake sample.

Extraction yield was calculated using the following equation:

(3) Extraction Yield(%)=(DEW/CSW)×100,

where CSW is the weight of the cake sample used for extraction. Unless otherwise noted, extraction yields are expressed on a dry-weight basis.

### 2.6. Determination of TFC

TFC was quantified using a colorimetric assay adapted from [18] with minor modifications. Briefly, 10 μL of each MPC extract prepare as described in Section 2.5 was added to a reaction mixture containing 60 μL of deionized water and 30 μL of 5% (*w*/*v*) sodium nitrite (NaNO_2_). After standing for 6 min at room temperature, 25 μL of 2.5% (*w*/*v*) aluminum chloride (AlCl_3_) solution, 50 μL of deionized water, and 25 μL of 2% (*w*/*v*) sodium hydroxide (NaOH) were added sequentially. The mixture was vortexed gently and allowed to react for 15 min at room temperature. Absorbance was measured at 415 nm using a UV-Vis spectrophotometer.

A standard calibration curve was prepared using quercetin solutions ranging from 31.25 to 2000 μg/mL. The calibration curve was linear with R^2^ = 0.9961. TFC was calculated from the standard curve and expressed as μg quercetin equivalents (QE) per gram of dry sample (μg QE/g).

Flavonoid content was therefore quantified using the widely adopted NaNO_2_-AlCl_3_ colorimetric method with quercetin calibration, following procedures similar to those reported for pomegranate fruit extract nanophytosomes [16].

### 2.7. Antioxidant Capacity Analysis

All antioxidant assays were conducted using solvent extracts prepared as described in Section 2.5.

#### 2.7.1. Determination of DPPH Free Radical Scavenging Capacity

DPPH radical scavenging activity of MPC extracts was determined according to [19] with minor modifications. Briefly, 1.0 mL of the extract was mixed with 3.0 mL of 0.2 mM DPPH methanolic solution. The mixture was vortexed and allowed to stand in darkness at room temperature for 30 min. Absorbance was measured at 517 nm using a UV-Vis spectrophotometer. Lower absorbance indicates stronger radical scavenging activity. Results were quantified using a Trolox calibration curve and expressed as micrograms of Trolox equivalents (TE) per gram of sample (μg TE/g).

#### 2.7.2. Determination of ABTS Radical Scavenging Capacity

ABTS radical cation (ABTS•^+^) scavenging activity was measured following [20] with modifications. The ABTS•^+^ working solution was prepared by combining 1.5 mL deionized water, 0.25 mL peroxidase (44 U/mL), 0.25 mL of 500 μM H_2_O_2_, and 0.25 mL of 1000 μM ABTS, followed by incubation in the dark at room temperature for 1 h. For analysis, 1.5 mL of the freshly prepared ABTS•^+^ solution was mixed with 0.05 mL of the extract, and absorbance was recorded at 734 nm. Reduced absorbance corresponds to higher ABTS•^+^ scavenging activity. Values were calculated from a Trolox standard curve and reported as μg TE/g.

#### 2.7.3. Determination of FICA

FICA was assessed using the method of [21] with slight modifications. A 0.5 mL aliquot of extract was mixed with 0.5 mL of 2 mM FeCl_2_ solution. The reaction was initiated by adding 0.2 mL of 5 mM ferrozine, followed by vortexing. The mixture was incubated for 10 min at room temperature to allow formation of the ferrozine-Fe^2+^ complex. Absorbance was measured at 562 nm. Lower absorbance indicates stronger chelating activity due to inhibition of complex formation. Results were calculated using an ethylenediaminetetraacetic acid (EDTA) standard curve and expressed as μg EDTA equivalents (EDTAE) per gram of sample (μg EDTAE/g).

#### 2.7.4. Determination of FRAP

FRAP was determined following [22] with minor modifications. The FRAP working reagent was freshly prepared by mixing acetate buffer (300 mM, pH 3.2), 10 mM 2,4,6-tris(2′-pyridyl)-1,3,5-triazine (TPTZ) solution in 40 mM HCl, and 20 mM FeCl_3_·6H_2_O at a 10:1:1 (*v*/*v*/*v*) ratio. For analysis, 30 μL of extract or standard was mixed with 900 μL of FRAP reagent and vortexed, followed by addition of 90 μL deionized water. The mixture was incubated at 37 °C for 60 min, and absorbance was measured at 593 nm. A standard curve was prepared using L-ascorbic acid (15.625–1000 ppm), and FRAP values were expressed as μg vitamin C equivalents (VCE) per gram of sample (μg VCE/g).

#### 2.7.5. RP Assay

RP was determined using the method of [23] with modifications. A 300 μL aliquot of extract was mixed with 300 μL phosphate-buffered saline (PBS, pH 7.0) and 300 μL of 1% potassium ferricyanide solution. The mixture was incubated at 50 °C for 20 min and cooled rapidly in an ice bath. Next, 300 μL of 10% trichloroacetic acid (TCA) was added, and the mixture was centrifuged at 3000 rpm for 10 min. A 500 μL aliquot of the supernatant was combined with 500 μL deionized water, followed by 100 μL of 0.1% FeCl_3_·6H_2_O. After 10 min at room temperature, absorbance was measured at 700 nm. A calibration curve using L-ascorbic acid (15.625–1000 ppm) was used to calculate RP, and results were expressed as μg VCE/g.

### 2.8. Statistical Analysis

Unless otherwise stated, all results are expressed as mean ± standard deviation (SD) from at least three independent replicates (n = 3). Statistical analyses were performed using two separate comparison frameworks due to differences in the experimental design.

Comparisons between LGF and MPP were carried out using an independent-samples *t*-test, as these raw ingredients represent two distinct materials not included within the experimental substitution matrix.

For chiffon cake samples, MPC0–MPC80, the effects of MPP substitution on TPC, TFC, extraction yield, and antioxidant indices, DPPH, ABTS, FRAP, FICA, RP, were evaluated using one-way ANOVA conducted separately for each response variable and extraction solvent. When a significant main effect was detected (*p* < 0.05), Tukey’s honestly significant difference (HSD) test was applied for post hoc pairwise comparisons.

The separation of analyses for LGF/MPP and MPC0–MPC80 was necessary because raw ingredients cannot be included in the same ANOVA model as baked formulations, due to differences in treatment structure, matrix characteristics, and variance patterns.

Pearson’s correlation coefficients (r) were calculated to assess relationships between phenolic parameters, free phenolics, bound phenolics, TPC, TFC, and antioxidant indices using mean values from all cake–solvent combinations. Data normality and homogeneity of variance were verified using the Shapiro–Wilk and Levene tests, respectively. All statistical analyses were conducted using the Statistica 14.0 (TIBCO Software Inc., Palo Alto, CA, USA).

## 3. Results and Discussion

### 3.1. Effect of Mycoprotein Substitution on the TPC of Chiffon Cakes

Phenolic compounds in food matrices exist as free phenolics, which are soluble and readily extracted by aqueous-organic solvents, and bound phenolics, which are covalently linked to cell wall polysaccharides, dietary fiber, or proteins. Consistent with this structural distinction, free phenolics can be directly extracted, whereas bound phenolics require alkaline or acid hydrolysis for release [24]. TPC represents the sum of these fractions and reflects the overall abundance of polyphenolic antioxidants in a food system.

The chiffon cakes formulated with varying levels of MPP exhibited significant differences in phenolic distribution (Table 2). Across all formulations, the bound phenolic fraction exceeded the free fraction, indicating that a substantial proportion of phenolics were associated with structural macromolecules such as β-glucans, chitin, and cell wall proteins [3,11]. In the control sample (MPC0), free phenolics were 583.65 μg GAE/g, whereas bound phenolics reached 1263.48 μg GAE/g, together giving a total phenolic content (TPC) of 1847.13 μg GAE/g, with bound phenolics accounting for approximately 68% of TPC. With increasing MPP substitution, both free and bound phenolic contents rose significantly (*p* < 0.05), and total phenolics increased approximately 82% from MPC0 to MPC80. At the highest substitution level (MPC80), free phenolics increased to 837.57 μg GAE/g and bound phenolics to 2528.70 μg GAE/g, yielding a TPC of 3366.27 μg GAE/g, of which bound phenolics represented approximately 75%. These results indicate that MPP contributes substantially to the bound phenolic pool, consistent with reports that fungal cell walls are rich in phenolic acids that remain structurally bound until released through hydrolysis or processing [10,24].

The phenolic composition of the raw ingredients supports this distribution pattern. LGF contained 1201 μg GAE/g of free phenolics and 772 μg GAE/g of bound phenolics, whereas MPP exhibited markedly higher levels of both, including approximately 1448 μg GAE/g free phenolics and 7946 μg GAE/g bound phenolics. Bound phenolics therefore represented nearly 85% of the total phenolics in MPP, consistent with previous studies showing that fungal biomass contains phenolic acids such as gallic, protocatechuic, and *p*-hydroxybenzoic acids esterified or ether-linked to chitin, β-glucans, and proteins [3,11,25].

The baking process may also influence phenolic extractability and distribution. Heat treatment and the mechanical disruption of cake structure during mixing and rising can partially degrade fungal cell walls, causing release of structurally trapped phenolic compounds [9,25]. This phenomenon explains the simultaneous increases in both free and bound phenolics observed in cakes enriched with MPP. Previous research on cereal- and mushroom-based foods has similarly reported that thermal processing enhances the release of bound phenolics and increases their measured antioxidant activity [9,25].

The coexistence of free and bound phenolics has important implications for antioxidant behavior in chiffon cake. Free phenolics rapidly donate hydrogen atoms or electrons and contribute to immediate radical-scavenging activity, while bound phenolics become bioactive only after hydrolysis or digestion and therefore function as delayed-release antioxidants [24]. The higher proportion of bound phenolics in MPP-substituted cakes suggests that these products may provide both rapid antioxidant activity and prolonged protective effects during digestion.

In summary, partial replacement of LGF with MPP significantly increased free, bound, and TPC in chiffon cake. The marked increase in bound phenolics reflects the contribution of fungal cell wall-associated phenolic acids, while the increase in free phenolics indicates improved extractability during processing. These results demonstrate that MPP enhances the phenolic profile of chiffon cakes and provides a foundation for improved antioxidant functionality in subsequent analyses.

### 3.2. Extraction Yield of Bioactive Compounds from Chiffon Cakes Using Solvents with Different Polarities

The extraction yield represents the proportion of solvent-soluble constituents released from the chiffon cake matrix and is influenced by both solvent polarity and the intrinsic chemical composition of the sample. As shown in Table 3, substitution of LGF with MPP produced a significant and progressive increase in extraction yield across all extraction solvents (*p* < 0.05). Under water extraction, yields increased from 15.80% in MPC0 to 27.36% in MPC80; with 50% ethanol, yields increased from 19.27% to 28.69%; and with 95% ethanol, yields rose from 10.18% to 21.26%. These findings indicate that MPP contributes a greater quantity of solvent-extractable material than LGF, consistent with its higher levels of phenolics, flavonoids, soluble fiber, proteins, and secondary metabolites [3,5,11].

Across all formulations, 50% ethanol achieved the highest extraction yields, followed by water and then 95% ethanol. This pattern is characteristic of matrices containing a mixture of moderately polar phenolic acids and flavonoid derivatives, which are most efficiently recovered in aqueous ethanol systems. The observed trend corresponds with established extraction behavior of fungal-derived polyphenols, which typically exhibit maximal solubility in alcohol-water mixtures of intermediate polarity [17].

Additional support for the effect of solvent polarity is provided by the extraction behavior of the raw ingredients. When extracted with 50% ethanol, MPP produced an extraction yield of 12.02%, exceeding that of LGF (8.98%). Filamentous fungal materials such as *F. venenatum* are known to contain diverse bioactive metabolites, including phenolic acids, flavonoid-like compounds, terpenoids, sterols, and metal-chelating constituents, many of which are preferentially solubilized in aqueous alcohol rather than in water alone or in low-polarity solvents [3,5,11]. The reduced performance of 95% ethanol reflects its limited ability to dissolve hydrophilic phenolics, polysaccharides, and amino-acid-derived metabolites, whereas the intermediate yields from water extraction suggest that some hydrophobic or moderately hydrophobic constituents require organic co-solvents for effective recovery.

The predominance of bound phenolics and the stronger increase in bound relative to free phenolics at higher MPP levels suggest that thermal processing and aeration of the chiffon cake matrix facilitate the release of cell wall-associated compounds. The β-glucan- and chitin-rich cell wall of *F. venenatum* can entrap phenolic acids and flavonoids in ester- or ether-linked forms, which are progressively liberated by heat, mechanical shear, and alkaline hydrolysis [10,25]. Baking at elevated temperature may disrupt these structural linkages and increase the accessibility of phenolics to extraction, while Maillard reaction products formed between reducing sugars and mycoprotein-derived amino acids can further modify the redox environment and potentially stabilize certain phenolic species. Together, these effects likely contribute to the greater extractable TPC observed in cakes with higher MPP substitution.

Overall, the progressive increase in extraction yield with higher MPP substitution provides initial evidence that bioactive constituents associated with antioxidant activity become increasingly accessible as fungal biomass replaces LGF. Together with the observed increases in TPC, these findings indicate enhanced extractability of phenolic acids and other soluble antioxidants in MPP-enriched chiffon cakes. The consistently superior performance of 50% ethanol further supports the interpretation that the principal extractable antioxidants in MPP possess moderate polarity and are aligned with previously reported chemical profiles of fungal cell wall-associated phenolics.

### 3.3. Effect of Partial Replacement of LGF with MPP on TFC in Chiffon Cake

Flavonoids constitute a major class of polyphenolic secondary metabolites and include flavones, flavanols, flavonols, flavanones, isoflavones, and anthocyanins. More than ten thousand flavonoid structures have been identified in natural sources [26]. These compounds are widely distributed in plants and microorganisms and are also present in fungal matrices, where they contribute to antioxidant, antimicrobial, and reducing activities. Previous studies have reported detectable flavonoids and structurally related metabolites in filamentous fungi such as *F. venenatum*, *Aspergillus oryzae*, and *Pleurotus ostreatus*, with demonstrated radical-scavenging and ferric-reducing activities [5,11]. Given that many flavonoids exhibit moderate thermal stability, a portion of these compounds can persist through baking. Accordingly, the inclusion of MPP in chiffon cake formulations is expected to enhance the flavonoid content and contribute to the antioxidant potential of the finished product.

The TFC of the raw ingredients supports this expectation. As shown in Table 4, MPP exhibited substantially higher TFC under 50% ethanol extraction (84.39 μg QE/g) compared with LGF (16.90 μg QE/g). This 5-fold difference reflects the richer reservoir of secondary metabolites associated with fungal biomass, consistent with previous reports describing the abundance of phenolic acids, flavonoids, and ergosterol-related compounds in mycoprotein materials [3,5].

The chiffon cakes prepared with graded substitution of LGF by MPP demonstrated clear, progressive increases in TFC across all extraction solvents. Under 50% ethanol extraction, TFC increased from 12.06 μg QE/g in MPC0 to 72.85 μg QE/g in MPC80, representing an approximately 6-fold rise. Water extraction produced an 8-fold increase (3.00 to 25.33 μg QE/g), whereas 95% ethanol extraction yielded a 14-fold increase (0.89 to 12.68 μg QE/g). These patterns reflect the polarity-dependent solubility of flavonoid subclasses, with aqueous ethanol most effectively extracting phenolics of moderate polarity typical of fungal cell wall-associated metabolites [17].

The substantial increases in TFC observed with MPP incorporation indicate that mycoprotein serves as an effective flavonoid-enhancing ingredient in chiffon cake. This outcome aligns with prior studies in which fungal powders or mushroom-derived materials increased flavonoid and phenolic content in baked products [13,27]. Because flavonoids participate in hydrogen-donating, electron-transfer, and metal-chelating reactions, the elevated TFC in MPP-enriched formulations likely contributes to the improved antioxidant performance observed in subsequent analyses.

The TPC values observed in the present study are within the range reported for other phenolic-enriched food systems analyzed by comparable Folin–Ciocalteu procedures. For example, the pomegranate fruit extract nanophytosomes described by [16] exhibited high TPC (371.19 ± 8.12 mg GAE/g dry weight) and TFC (194.97 ± 0.01 mg/100 g dry weight) values when quantified using the same gallic acid and quercetin calibration systems as applied here. While direct numerical comparison is limited by differences in matrix (encapsulated extract versus cake crumb), both studies show that increasing the proportion of a phenolic-rich ingredient (pomegranate extract or mycoprotein powder) yields a proportional enhancement in TPC, TFC, and antioxidant indicators.

Overall, the results demonstrate that partial substitution of LGF with MPP markedly enhances the flavonoid profile of chiffon cake and supports the potential of fungal-derived ingredients as functional components for antioxidant-oriented bakery reformulation.

### 3.4. Antioxidant Capacity of Chiffon Cake with Partial Replacement of LGF by MPP

Antioxidant function in food systems involves several reaction pathways, including hydrogen or electron donation, free-radical quenching, ferric-ion reduction, and metal-ion chelation [6]. To evaluate how MPP influences these mechanisms in chiffon cake, solvent extracts prepared with water, 50% ethanol, and 95% ethanol were analyzed using DPPH radical-scavenging, ABTS radical-scavenging, FRAP, FICA, and RP assays.

#### 3.4.1. DPPH Radical-Scavenging Capacity

The DPPH radical-scavenging assay evaluates the hydrogen-donating ability of antioxidants through the reduction of the stable DPPH radical, which produces a measurable decrease in absorbance [28]. In this study, DPPH radical-scavenging activity was quantified using a Trolox calibration curve.

**Figure 1:**
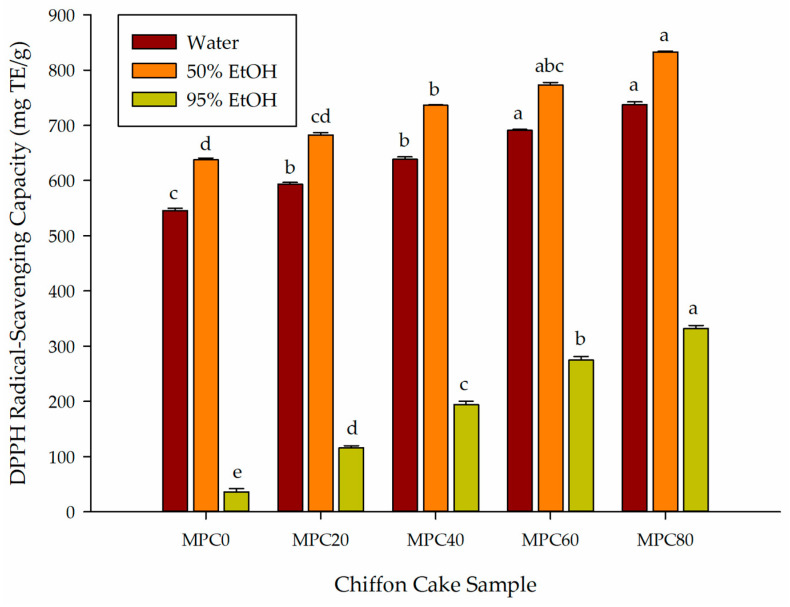
Effect of partial replacement of LGF with MPP on the DPPH radical-scavenging activity of extracted bioactive compounds from chiffon cake using solvents of different polarities. Values are presented as mean ± standard deviation (*n* = 3). For chiffon cake samples (MPC0–MPC80), different lowercase superscript letters (^a–^^e^) within each color indicate significant differences according to one-way ANOVA followed by Tukey’s honestly significant difference (HSD) test (*p* < 0.05). Abbreviations: MPC0 denotes chiffon cake without MPP; MPC20, MPC40, MPC60, and MPC80 denote chiffon cakes containing 20%, 40%, 60%, and 80% MPP, respectively; DPPH, 2,2-diphenyl-1-picrylhydrazyl; TE, Trolox equivalent.

As shown in Figure 1, partial replacement of LGF with MPP significantly increased the DPPH radical-scavenging capacity of chiffon cake extracts (*p* < 0.05). Across all three extraction solvents, DPPH radical-scavenging activity rose progressively with higher MPP substitution, corresponding to the increases observed in TPC (Section 3.1) and TFC (Section 3.3). Among the extraction solvents, 50% ethanol produced the strongest responses, increasing from 637.44 μg TE/g in MPC0 to 832.79 μg TE/g in MPC80. Water extracts showed a similar upward trend (545.58 to 737.44 μg TE/g). Although absolute values were lower in 95% ethanol extracts, this solvent produced the largest fold-increase (36.28 to 331.63 μg TE/g, approximately 9-fold), suggesting meaningful contributions from less-polar constituents in MPP.

These solvent-dependent patterns are consistent with prior findings that *F. venenatum* contains phenolic acids, flavonoid-like structures, and sterol-derived metabolites with strong radical-scavenging properties [11,29]. Additional studies have shown that alkaline disruption enhances DPPH activity in *F. venenatum* by releasing cell wall-bound phenolics [10], which supports the trends observed here across solvents of differing polarity. The large difference between the DPPH radical-scavenging activity of MPP itself (18,286 μg TE/g in 50% ethanol) and that of the MPC80 cake extract (832.79 μg TE/g) likely reflects partial degradation of heat-labile antioxidants during baking, as well as matrix interactions, including potential binding between phenolics and proteins or carbohydrates during Maillard and thermal reactions.

These results agree with studies on mushroom- or mycelium-enriched baked goods, which have similarly reported increased DPPH radical-scavenging activity following incorporation of fungal biomass [12]. Collectively, the evidence indicates that MPP supplies a diverse pool of radical-scavenging compounds and, despite processing losses, meaningfully enhances the antioxidant capacity of chiffon cake.

#### 3.4.2. ABTS Radical-Scavenging Activity

ABTS radical-scavenging assay measures the ability of antioxidant compounds to reduce the blue-green ABTS•^+^ generated from 2,2′-azino-bis-(3-ethylbenzothiazoline-6-sulfonic acid). Antioxidant-mediated reduction of ABTS•^+^ produces a decline in absorbance, providing an index of radical-scavenging activity and electron-donating capacity [28]. ABTS activity in this study was quantified using a Trolox calibration curve.

**Figure 2:**
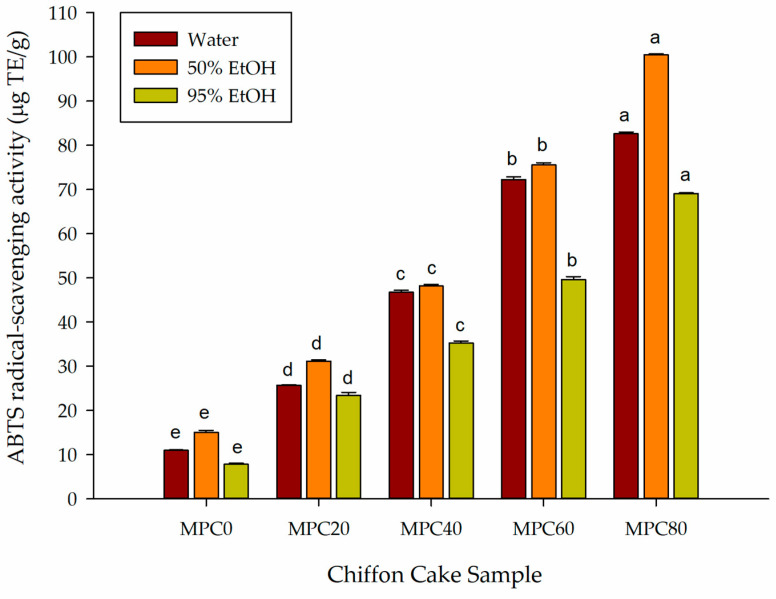
Effect of partial replacement of LGF with MPP on the ABTS radical-scavenging activity of bioactive compounds from chiffon cake using solvents of different polarities. Values are presented as mean ± standard deviation (*n* = 3). For chiffon cake samples (MPC0–MPC80), different lowercase superscript letters (^a–e^) within each color indicate significant differences according to one-way ANOVA followed by Tukey’s honestly significant difference (HSD) test (*p* < 0.05). Abbreviations: ABTS, 2,2′-azino-bis-(3-ethylbenzothiazoline-6-sulfonic acid); TE, Trolox equivalent; MPC0 denotes chiffon cake without MPP; MPC20, MPC40, MPC60, and MPC80 denote chiffon cakes containing 20%, 40%, 60%, and 80% MPP, respectively.

As shown in Figure 2, substitution of LGF with MPP led to a significant and progressive enhancement of ABTS radical-scavenging activity across all solvent systems (*p* < 0.05). Consistent with the polarity-dependent behavior observed in raw ingredient extracts, the 50% ethanol extract of MPP exhibited the strongest ABTS activity (231.41 μg TE/g), followed by the water extract (177.38 μg TE/g). This solvent hierarchy reflects the predominance of moderately polar phenolic acids and flavonoids in fungal biomass and aligns with prior reports that ethanol-based fungal extracts often outperform aqueous extracts in ABTS radical-scavenging assays [30]. Additional findings from alkaline-treated *F. venenatum* demonstrate that wall-bound phenolics are liberated upon cell wall disruption and contribute significantly to ABTS quenching [10].

In chiffon cake formulations, ABTS radical-scavenging activity increased substantially with higher MPP substitution. Water extracts rose from 10.98 μg TE/g in MPC0 to 82.59 μg TE/g in MPC80 (approximately 7.5-fold). Under 50% ethanol, the values increased from 15.01 to 100.46 μg TE/g (approximately 6.7-fold), while 95% ethanol extracts increased from 7.81 to 69.05 μg TE/g (approximately 9-fold). The strongest activity in MPC80 was obtained with 50% ethanol, demonstrating that moderately polar constituents from MPP are most efficiently extracted under intermediate-polarity conditions.

The consistent increases across all three solvents indicate that MPP contributes a broad spectrum of hydrophilic and hydrophobic antioxidant compounds to the chiffon cake matrix. These findings parallel observations that mushroom- and mycelium-derived powders enhance ABTS, DPPH, and FRAP antioxidant performance in baked goods [8]. Overall, the ABTS results confirm that partial replacement of LGF with MPP meaningfully enhances the radical-scavenging capacity of chiffon cake, reflecting the increased availability of phenolic and flavonoid constituents contributed by the fungal ingredient.

#### 3.4.3. FRAP Value

The FRAP assay measures the electron-donating ability of antioxidant compounds by quantifying their capacity to reduce ferric ions (Fe^3+^) to ferrous ions (Fe^2+^). In an acidic medium, Fe^3+^ forms a colorless complex with TPTZ, which is converted to the blue Fe^2+^(TPTZ)_2_ complex upon reduction; the increase in absorbance at 593 nm provides an index of reducing capacity [28]. In this study, vitamin C was used as the calibration standard.

**Figure 3:**
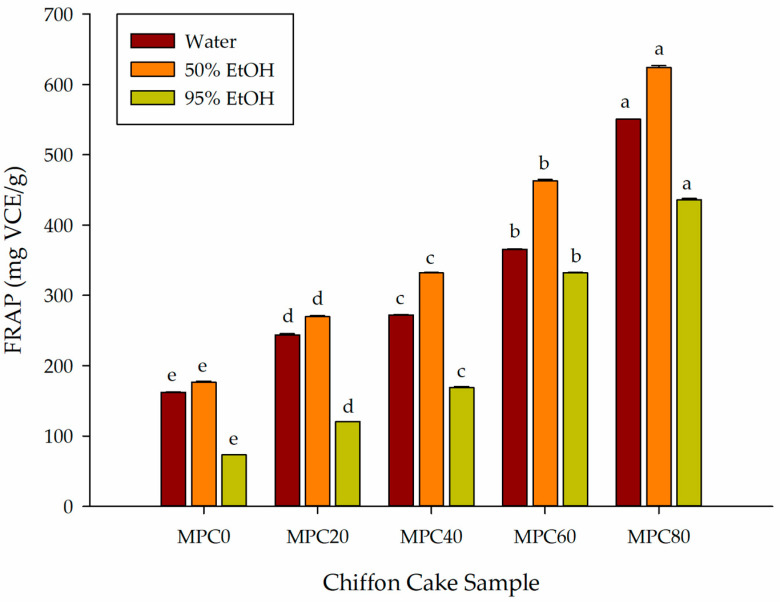
Effect of partial replacement of LGF with MPP on the FRAP of extracted bioactive compounds from chiffon cake using solvents of different polarities. Values are presented as mean ± standard deviation (*n* = 3). For chiffon cake samples (MPC0–MPC80), different lowercase superscript letters (^a–e^) within each color indicate significant differences according to one-way ANOVA followed by Tukey’s honestly significant difference (HSD) test (*p* < 0.05). Abbreviations: FRAP, ferric reducing antioxidant power; VCE, vitamin C equivalents; MPC0 denotes chiffon cake without MPP; MPC20, MPC40, MPC60, and MPC80 denote chiffon cakes containing 20%, 40%, 60%, and 80% MPP, respectively.

As shown in Figure 3, FRAP values increased significantly with greater substitution of LGF by MPP across all extraction solvents (*p* < 0.05). Under 50% ethanol extraction, FRAP increased from 176.82 μg VCE/g in MPC0 to 624.11 μg VCE/g in MPC80. Water extracts showed a similar progression, rising from 162.09 to 550.47 μg VCE/g, while 95% ethanol extracts increased from 73.33 to 435.74 μg VCE/g. These trends indicate that the inclusion of MPP substantially enhances the ferric-reducing capacity of chiffon cake, consistent with the increases observed in TPC and TFC.

Across all formulations, 50% ethanol produced the highest FRAP values, reflecting the predominance of moderately polar antioxidant compounds in fungal biomass. This pattern parallels the solvent-dependent behavior observed for TPC and TFC, both of which were maximized in 50% ethanol extracts. The enhanced FRAP likely derives from phenolic acids, flavonoids, and other redox-active metabolites associated with fungal cell wall structures and secondary metabolic pathways.

The strong ferric-reducing activity of MPP corresponds with prior reports describing fungal materials as rich sources of phenolic acids, peptides, and polysaccharides capable of donating electrons and enhancing reducing capacity [10]. Additional evidence indicates that ethanol extracts of fungal biomass typically exhibit stronger FRAP activity compared with aqueous and highly alcoholic solvents [30], supporting the solvent-polarity relationships observed in this study.

Overall, the progressive elevation of FRAP values with increasing MPP incorporation demonstrates a substantial enhancement of reducing capacity in chiffon cake and highlights the contribution of fungal-derived phenolics and flavonoids to its antioxidant functionality.

#### 3.4.4. FICA Value

The FICA assay evaluates the capacity of antioxidant compounds to bind ferrous ions (Fe^2+^), thereby limiting their involvement in Fenton-type reactions that generate highly reactive hydroxyl radicals. By forming stable complexes with Fe^2+^, chelating agents can reduce metal-catalyzed oxidative deterioration and contribute meaningfully to the overall antioxidant performance of food matrices.

**Figure 4:**
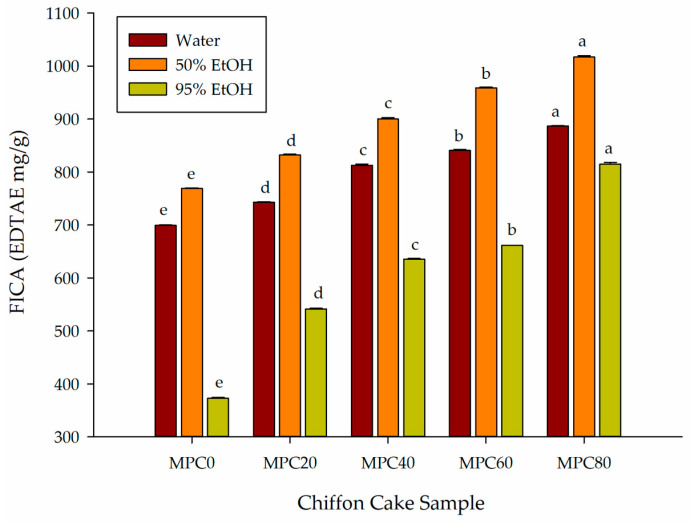
Effect of partial replacement of LGF with MPP on the FICA value of extracted bioactive compounds from chiffon cake using solvents of different polarities. Values are presented as mean ± standard deviation (*n* = 3). For chiffon cake samples (MPC0–MPC80), different lowercase superscript letters (^a–e^) within each color indicate significant differences according to one-way ANOVA followed by Tukey’s honestly significant difference (HSD) test (*p* < 0.05). Abbreviations: FICA, ferrous ion-chelating activity; EDTAE, ethylenediaminetetraacetic acid equivalent; MPC0 denotes chiffon cake without MPP; MPC20, MPC40, MPC60, and MPC80 denote chiffon cakes containing 20%, 40%, 60%, and 80% MPP, respectively.

As shown in Figure 4, partial substitution of LGF with MPP significantly increased the FICA of chiffon cake extracts across all solvent systems (*p* < 0.05). In water extracts, FICA values increased from 699 μg EDTAE/g in MPC0 to 886 μg EDTAE/g in MPC80. In 50% ethanol extracts, values rose from 769 to 1017 μg EDTAE/g, while 95% ethanol extracts increased from 373 to 815 μg EDTAE/g. Among all treatments, the MPC80 sample extracted with 50% ethanol exhibited the highest chelating activity, indicating that moderately polar constituents of MPP contributed most effectively to Fe^2+^ sequestration.

These findings are consistent with earlier reports that fungal materials possess strong metal-chelating activity due to their diverse composition of phenolic acids, flavonoids, and polysaccharide- or protein-linked functional groups [31]. Chelation typically arises from structural features such as catechol and pyrogallol moieties in phenolic acids, ortho-dihydroxyl groups in flavonoids, and carboxyl or ester functionalities found in fungal polysaccharides and proteins, all of which can form coordination complexes with Fe^2+^. Similar observations have been documented for mushroom-derived ingredients used in baked products, where the incorporation of fungal powders enhances the metal-chelating performance of the final product [12,30].

The progressive increase in the FICA value with greater MPP incorporation suggests that mycoprotein contributes a substantial pool of metal-binding compounds to the chiffon cake matrix. This enhanced chelating activity represents an important mechanism by which MPP improves the oxidative stability and antioxidant functionality of fortified chiffon cakes, complementing its contributions to radical scavenging and ferric-reducing capacity.

#### 3.4.5. RP Value

The RP value reflects the ability of antioxidant compounds to donate electrons and convert oxidized intermediates into more stable and less reactive forms. Because electron donation is a fundamental antioxidant mechanism in food systems, the RP assay provides an important index of the redox activity of bioactive constituents. Fungal materials, including mycoprotein, are known to contain phenolic acids, flavonoids, sterol derivatives, and other redox-active metabolites that contribute significantly to electron-donating capacity [3,4,11,29].

**Figure 5:**
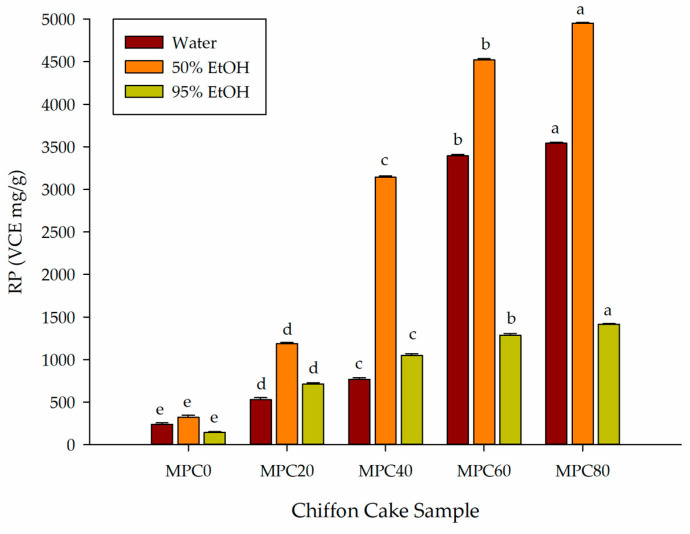
Effect of partial replacement of LGF with MPP on the RP value of extracted bioactive compounds from chiffon cake using solvents of different polarities. Values are presented as mean ± standard deviation (*n* = 3). For chiffon cake samples (MPC0–MPC80), different lowercase superscript letters (^a–e^) within each color indicate significant differences according to one-way ANOVA followed by Tukey’s honestly significant difference (HSD) test (*p* < 0.05). Abbreviations: RP = reducing power; VCE, vitamin C equivalents; MPC0 denotes chiffon cake without MPP; MPC20, MPC40, MPC60, and MPC80 denote chiffon cakes containing 20%, 40%, 60%, and 80% MPP, respectively.

As shown in Figure 5, the RP of chiffon cake extracts increased markedly with higher levels of MPP substitution across all solvent systems (*p* < 0.05). In water extracts, RP increased from 239 μg VCE/g in MPC0 to 3541 μg VCE/g in MPC80, representing approximately a 14-fold increase. The 50% ethanol extracts exhibited the highest overall response, rising from 324 to 4953 μg VCE/g (approximately 15-fold). Extracts prepared with 95% ethanol also increased substantially, from 145 to 1416 μg VCE/g (approximately 9.8-fold). The superior performance of the 50% ethanol extracts is consistent with the behavior of moderately polar metabolites, which constitute a major portion of the antioxidant constituents in fungal biomass.

These results correspond closely with earlier findings that fungal-derived ingredients can substantially enhance the RP of baked products by enriching the matrix with phenolics, flavonoids, and other electron-donating compounds [13]. The substantial increases in RP in MPP-enriched samples, particularly at the MPC80 level, suggest that although some heat-sensitive components may be lost during baking, a large proportion of the redox-active constituents in mycoprotein remain functional in the final product.

Overall, the observed increases in the RP value confirm that MPP substantially enhances the electron-donating antioxidant mechanisms of chiffon cake. When considered alongside the improvements observed in DPPH and ABTS radical-scavenging capacity, FRAP, and FICA, the strengthened RP value highlights the multifaceted antioxidant benefits conferred by mycoprotein incorporation.

### 3.5. Correlation Analysis of Phenolic Compounds and Antioxidant Indices

Fungal-derived materials contain a wide range of secondary metabolites, including phenolic acids, flavonoids, terpenoids, and sterol-related compounds, many of which exhibit well documented antioxidant properties [32]. Among these constituents, phenolic compounds are generally recognized as key contributors to antioxidant behavior because of their ability to donate electrons or hydrogen atoms and thereby neutralize reactive species [33]. To evaluate the role of these compounds in mycoprotein-enriched chiffon cake, Pearson correlation analysis was conducted to assess the relationships between TPC, TFC, and various antioxidant indices.

As shown in Table 5, total phenolic content (TPC) showed very strong positive correlations with all antioxidant indices. For example, TPC was highly correlated with DPPH radical-scavenging activity (r = 0.983, *p* < 0.01), ABTS activity (r ≈ 0.951, *p* < 0.01), and FRAP values (r ≈ 0.908, *p* < 0.01), indicating that increases in mycoprotein-derived phenolics were closely associated with enhanced radical-scavenging and ferric-reducing capacities. Total flavonoid content (TFC) also correlated strongly with ABTS activity and FRAP values (e.g., r > 0.95, *p* < 0.01), although the slightly lower coefficients for TFC versus DPPH suggest that non-flavonoid phenolics also contribute to overall antioxidant performance. In contrast, correlations involving bound phenolics were somewhat weaker than those for free phenolics, supporting the interpretation that readily extractable phenolics play a dominant role in the measured in vitro antioxidant responses.

TFC also displayed significant positive correlations with ABTS activity, FRAP values, and RP values, suggesting that flavonoids play an important complementary role in the antioxidant performance of chiffon cake extracts. The slightly weaker correlation between TFC and DPPH activity may be attributed to the differential reactivity of specific flavonoid subclasses toward the DPPH radical.

In contrast, bound phenolics showed comparatively weaker relationships with most antioxidant indices, exhibiting a notable correlation only with DPPH activity. This observation suggests that although bound phenolics contribute to antioxidant potential, their influence is less immediate than that of free phenolics, which are more readily extractable under the assay conditions used.

Our observations are consistent with reports that incorporating mushroom or fungal powders into bakery products increases TPC and antioxidant activity. For example, Ref. [34] observed an approximate 2-fold increase in DPPH scavenging and FRAP values in bread enriched with Pleurotus mushroom powder, and ref. [35] reported elevated TPC and ABTS activity in wheat bread fortified with *Ganoderma lucidum* extract. The approximate 82% increase in TPC and multi-fold increases in DPPH, ABTS, FRAP, and RP values observed at 80% MPP substitution in the present study are comparable to, or greater than, these enhancements, indicating that mycoprotein performs at least as well as other fungal ingredients in improving the antioxidant potential of baked products.

These findings are consistent with earlier reports demonstrating strong associations between phenolic composition and antioxidant behavior in both fungal materials and plant-based foods [7,13]. Overall, the correlation analysis confirms that the improved antioxidant capacity of chiffon cakes fortified with MPP is primarily driven by increases in total phenolics and flavonoids, especially the free phenolic fraction, underscoring the functional significance of mycoprotein as a bioactive ingredient in bakery applications.

Although the current work focuses on compositional and in vitro antioxidant endpoints, future studies should examine the bioaccessibility and bioavailability of mycoprotein-derived phenolics during digestion and assess whether the enhanced antioxidant capacity translates into improved oxidative stability and shelf-life of chiffon cake under storage.

## 4. Conclusions

This study demonstrates that substituting low-gluten flour with mycoprotein powder substantially improves the phenolic composition and antioxidant functionality of chiffon cakes. Increasing mycoprotein levels increased total phenolics, flavonoids, and free phenolic fractions, which was reflected in higher DPPH and ABTS radical-scavenging capacities, more ferric reducing antioxidant power, increased ferrous ion-chelating ability, and higher reducing power. These compositional and functional enhancements, combined with previously reported acceptable texture and sensory properties, position mycoprotein the chiffon cake as a promising age-friendly bakery product for young children, older adults, and health-conscious consumers. Future work should evaluate antioxidant bioavailability in vitro and in vivo, assess shelf-life and oxidative stability during storage, and integrate more comprehensive consumer sensory studies to further clarify both health benefits and the market potential of mycoprotein-fortified chiffon cakes.

## Figures and Tables

**Table 1:** Formulation of chiffon cakes with different substitution levels of MPP for LGF.

Ingredient	MPC0	MPC20	MPC40	MPC60	MPC80
MPP (g)	0.00	20.00	40.00	60.00	80.00
LGF (g)	100.00	80.00	60.00	40.00	20.00
Baking powder (g)	1.00	1.00	1.00	1.00	1.00
Soybean oil (g)	66.00	66.00	66.00	66.00	66.00
Water (g)	70.00	70.00	70.00	70.00	70.00
Rum (g)	6.25	6.25	6.25	6.25	6.25
Sugar (1) (g)	6.25	6.25	6.25	6.25	6.25
Egg yolk (g)	114.00	114.00	114.00	114.00	114.00
Egg white (g)	200.00	200.00	200.00	200.00	200.00
Sugar (2) (g)	83.00	83.00	83.00	83.00	83.00
IMO (g)	16.00	16.00	16.00	16.00	16.00
Total (g)	662.50	662.50	662.50	662.50	662.50

Note: The total flour content (low-gluten flour + mycoprotein powder) was kept constant at 100 g in all formulations. Sugar (1) was added to the yolk batter (liquid) phase, whereas Sugar (2) was added gradually during egg-white whipping to prepare the meringue. MPP, mycoprotein powder; LGF, low-gluten flour; IMO, isomalto-oligosaccharide; MPC, mycoprotein chiffon cake. MPC0 denotes the control cake with 0% MPP, whereas MPC20, MPC40, MPC60, and MPC80 correspond to cakes in which 20%, 40%, 60%, and 80% of the total flour mass, respectively, were supplied by MPP. Formulation adapted from our previous work [14].

**Table 2:** Total phenolic content of chiffon cakes formulated with different proportions of MPP.

Replacement (%)	Phenol Content (μg GAE/g)		
	Free Phenol	Bound Phenol	Total Phenol
LGF	1201.04 ± 12.30 ^B^	772.17 ± 12.30 ^B^	1973.22 ± 0.01 ^B^
MPP	1448.00 ± 17.22 ^A^	7946.09 ± 36.89 ^A^	9394.09 ± 54.11 ^A^
MPC0	583.65 ± 9.84 ^c^	1263.48 ± 30.74 ^e^	1847.13 ± 40.58 ^e^
MPC20	641.04 ± 7.38 ^c^	1533.04 ± 18.45 ^d^	2174.09 ± 11.07 ^d^
MPC40	665.39 ± 7.38 ^c^	1741.74 ± 30.74 ^c^	2407.13 ± 38.12 ^c^
MPC60	745.39 ± 17.22 ^b^	2246.09 ± 18.45 ^b^	2991.48 ± 35.66 ^b^
MPC80	837.57 ± 9.84 ^a^	2528.70 ± 12.30 ^a^	3366.26 ± 22.14 ^a^

Note: Values are presented as mean ± standard deviation (*n* = 3). For LGF and MPP, different uppercase superscript letters (^A, B^) within each column indicate significant differences based on independent-samples *t*-tests (*p* < 0.05). For chiffon cake samples (MPC0–MPC80), different lowercase superscript letters (^a–e^) within each column indicate significant differences according to one-way ANOVA followed by Tukey’s honestly significant difference (HSD) test (*p* < 0.05). LGF and MPP were analyzed separately from MPC0–MPC80 because raw ingredients and baked products differ in matrix structure and variance patterns and cannot be included in the same ANOVA model. Abbreviations: MPP, mycoprotein powder; LGF, low-gluten flour; GAE, gallic acid equivalents. MPC0 denotes chiffon cakes without MPP; MPC20, MPC40, MPC60, and MPC80 denote chiffon cakes containing 20%, 40%, 60%, and 80% MPP, respectively.

**Table 3:** Extraction yield of bioactive compounds from chiffon cakes partially substituted with mycoprotein powder using solvents with different polarities.

Replacement (%)	Extraction Yield (%)		
	Water	50% EtOH	95% EtOH
LGF	7.54 ± 0.53 ^B^	8.98 ± 0.23 ^B^	2.10 ± 0.15 ^B^
MPP	11.40 ± 0.07 ^A^	12.02 ± 0.38 ^A^	9.24 ± 0.07 ^A^
MPC0	15.80 ± 0.92 ^e^	19.27 ± 0.82 ^c^	10.18 ± 0.57 ^e^
MPC20	18.09 ± 0.47 ^d^	21.05 ± 1.47 ^bc^	13.34 ± 0.19 ^d^
MPC40	21.79 ± 0.30 ^c^	23.63 ± 2.22 ^b^	16.79 ± 0.66 ^c^
MPC60	24.79 ± 1.26 ^b^	26.54 ± 0.19 ^a^	18.99 ± 0.09 ^b^
MPC80	27.36 ± 1.42 ^a^	28.69 ± 0.74 ^a^	21.26 ± 1.28 ^a^

Note: Values are presented as mean ± standard deviation (*n* = 3). For LGF and MPP, different uppercase superscript letters (^A, B^) within each column indicate significant differences based on independent-samples *t*-tests (*p* < 0.05). For chiffon cake samples (MPC0–MPC80), different lowercase superscript letters (^a–e^) within each column indicate significant differences according to one-way ANOVA followed by Tukey’s honestly significant difference (HSD) test (*p* < 0.05). LGF and MPP were analyzed separately from MPC0–MPC80 because raw ingredients and baked products differ in matrix structure and variance patterns and cannot be included in the same ANOVA model. Abbreviations: MPP, mycoprotein powder; LGF, low-gluten flour. MPC0 denotes chiffon cakes without MPP; MPC20, MPC40, MPC60, and MPC80 denote chiffon cakes containing 20%, 40%, 60%, and 80% MPP, respectively.

**Table 4:** Effect of partial replacement of LGF with MPP on the TFC of extracted bioactive compounds from chiffon cake using solvents of different polarities.

Replacement (%)	TFC (μg QE/g)		
	Water	50%EtOH	95%EtOH
LGF	5.48 ± 0.18 ^B^	16.90 ± 0.53 ^B^	3.25 ± 0.18 ^B^
MPP	39.35 ± 0.44 ^A^	84.39 ± 0.18 ^A^	32.41 ± 0.79 ^A^
MPC0	3.00 ± 0.18 ^e^	12.06 ± 0.44 ^e^	0.89 ± 0.09 ^d^
MPC20	4.74 ± 0.35 ^d^	39.60 ± 0.26 ^d^	3.50 ± 0.01 ^c^
MPC40	8.96 ± 0.35 ^c^	53.75 ± 0.44 ^c^	4.86 ± 0.26 ^c^
MPC60	12.80 ± 0.61 ^b^	65.29 ± 0.35 ^b^	7.72 ± 0.18 ^b^
MPC80	25.33 ± 0.18 ^a^	72.85 ± 0.44 ^a^	12.68 ± 0.18 ^a^

Note: Values are presented as mean ± standard deviation (*n* = 3). For LGF and MPP, different uppercase superscript letters (^A, B^) within each column indicate significant differences based on independent-samples *t*-tests (*p* < 0.05). For chiffon cake samples (MPC0–MPC80), different lowercase superscript letters (^a–e^) within each column indicate significant differences according to one-way ANOVA followed by Tukey’s honestly significant difference (HSD) test (*p* < 0.05). LGF and MPP were analyzed separately from MPC0–MPC80 because raw ingredients and baked products differ in matrix structure and variance patterns and cannot be included in the same ANOVA model. Abbreviations: MPP, mycoprotein powder; LGF, low-gluten flour; TFC, total flavonoid content; QE, quercetin equivalents. MPC0 denotes a chiffon cake without MPP; MPC20, MPC40, MPC60, and MPC80 denote chiffon cakes containing 20%, 40%, 60%, and 80% MPP, respectively.

**Table 5:** Pearson correlation matrix among phenolic components and antioxidant indices in chiffon cake formulated with mycoprotein powder (MPP).

	Bound Phenol	Free Phenol	TPC	TFC	DPPH	ABTS	FRAP	FICA	RP
Bound phenol	1.000								
Free phenol	0.700	1.000							
TPC	0.749	0.996 **	1.000						
TFC	0.546	0.873 *	0.848 *	1.000					
DPPH	0.793 *	0.971 **	0.983 **	0.75	1.000				
ABTS	0.693	0.961 **	0.951 **	0.966 **	0.888 **	1.000			
FRAP	0.676	0.916 **	0.908 **	0.975 **	0.826 *	0.988 **	1.000		
FICA	0.708	0.851 *	0.845 *	0.968 **	0.767 *	0.957 **	0.972 **	1.000	
RP	0.493	0.794 *	0.762 *	0.973 **	0.651	0.923 **	0.946 **	0.942 **	1.000

Note: Pearson correlation coefficients (r) were calculated using mean values from all cake–solvent combinations (*n* = 15). Correlation significance was evaluated using two-tailed tests. Superscript symbols indicate statistical significance: *, *p* < 0.05 and **, *p* < 0.01. All statistical analyses were performed using Statistica software (TIBCO Software Inc., Palo Alto, CA, USA). Abbreviations: TPC, total phenolic content; TFC, total flavonoid content; DPPH, 2,2-diphenyl-1-picrylhydrazyl; ABTS, 2,2′-azino-bis-(3-ethylbenzothiazoline-6-sulfonic acid); FRAP, ferric reducing antioxidant power; FICA, ferrous-ion chelating activity; RP, reducing power.

## Data Availability

The data that support the findings of this study are available from the corresponding authors upon reasonable request. The data are not publicly available due to project-related and institutional restrictions associated with the ongoing industry–academia collaboration between Hungkuang University and Grape King Bio Ltd.

## Abbreviations

The following abbreviations are used in this manuscript:

ABTS	2,2′-azino-bis-(3-ethylbenzothiazoline-6-sulfonic acid)
ABTS•^+^	ABTS radical cation
DEW	Dried Extract Weight
DPPH	2,2-diphenyl-1-picrylhydrazyl
EDTA	Ethylenediaminetetraacetic acid
EDTAE	EDTA equivalent
FICA	Ferrous-ion Chelating Activity
FRAP	Ferric Reducing Antioxidant Power
GAE	Gallic Acid Equivalents
LGF	Low-Gluten Flour
MPC	Mycoprotein Chiffon Cake
MPC0	Mycoprotein Chiffon Cake with 0% Mycoprotein Powder
MPC80	Mycoprotein Chiffon Cake with 80% Mycoprotein Powder
MPP	Mycoprotein Powder
PBS	Phosphate-Buffered Saline
QE	Quercetin Equivalents
ROS	Reactive Oxygen Species
RP	Reducing Power
RPM	Revolutions per Minute
SD	Standard Deviation
TCA	Trichloroacetic Acid
TE	Trolox Equivalents
TFC	Total Flavonoid Content
TPTZ	2,4,6-tris(2′-pyridyl)-1,3,5-triazine
TPC	Total Phenolic Content
UV-Vis	Ultraviolet-Visible (Spectrophotometry)
VCEs	Vitamin C Equivalents
